# Molecular characterisation of *Mycoplasma* species isolated from the genital tract of Dorper sheep in South Africa

**DOI:** 10.4102/jsava.v86i1.1199

**Published:** 2015-06-08

**Authors:** Habu A. Kalshingi, Anna-Mari Bosman, Johan Gouws, Moritz van Vuuren

**Affiliations:** 1 National Veterinary Research Institute, Vom, Nigeria; 2 Department of Veterinary Tropical Diseases, University of Pretoria, South Africa

## Abstract

Biochemical and molecular analysis were conducted on 34 strains of Mycoplasma species isolated between 2003 and 2009 from the genital tract of clinically healthy Dorper sheep and sheep with ulcerative vulvitis and balanitis. Earlier publications identified the causative agent as *Mycoplasma mycoides mycoides* large colony (MmmLC) and *Arcanobacterium pyogenes*. The aims of the study were to characterise Mycoplasma species isolated from the genital tract of Dorper sheep with polymerase chain reaction assay, cloning and gene sequencing. Basic Local Alignment Search Tool (BLAST) results revealed six predominant Mycoplasma species: *Mycoplasma arginini, Mycoplasma bovigenitalium, Arcanobacterium laidlawii*, MmmLC, Mycoplasma sp. ovine/caprine serogroup II and *M. canadense.* Sequencing of the 34 isolates were analysed using phylogenetic methods, and 18 (50%) were identified as *M. arginini* with 99% – 100% similarity to *M. arginini* from England and Sweden. Six isolates showed 99% similarity to *M. bovigenitalium* strains from Turkey and Germany. Two isolates had 99% similarity to an M. sp. ovine/caprine sero group II from the United Kingdom. BLAST for two isolates revealed 99% similarity to *Acholeplasma laidlawii* from India, another two were 99% similar to MmmLC strain from Sweden, two showed 98% similarity to Mycoplasma sp. Usp 120 from Brazil, and two isolates have a 97% – 99% similarity to M. mm. Jcv1 strain from the United States of America. Finally, one isolate showed similarity of 99% to *Mycoplasma canadense* strain from Italy. The findings support the hypothesis that ulcerative vulvitis and balanitis of Dorper sheep in South Africa (SA) is a multifactorial disease with involvement of different Mycoplasma species.

## Introduction

Mycoplasmas are prokaryotic micro-organisms belonging to the class *Mollicutes*, which lacks rigid cell walls. Their genomic size ranges from 500 bp to 1500 bp. They cause a wide variety of different diseases in small ruminants, in particular ulcerative balanitis and vulvitis that affects Dorper sheep in South Africa (SA). The disease causes serious economic losses to Dorper sheep breeders in SA. This venereal disease is characterised by erosion and ulceration of the glans penis and vulval labia of sheep and has been described in several countries (Kidanemariam *et al.* 2005).

In SA the disease was first encountered in the Calvinia district of the Northern Cape province in 1979, and later spread to other parts of the country such as the Free State, KwaZulu-Natal, Eastern Cape and Western Cape (Bath & De Wet 2000; Trichard *et al.* 1993). A high prevalence of the disease in Dorper sheep in SA has been reported (Gummow & Staley 2000). In the United Kingdom (UK) a similar disease in ewes, with clinical signs such as swollen, oedematous, congested vulvas and blood-stained fluid or reddish stringy mucous oozing from the external orifices, has been reported. Other signs included vulval scabs on the lower commissure with small vesicles and plaques on the posterior floor of the vagina (Martin & Aitken 2000).

Greig (2007) divided the causative agents of ulcerative balanitis and vulvitis in sheep flocks in the UK into four main entities: venereal parapoxvirus (orf) infection; enzootic posthitis (pizzle rot) caused by *Corynebactrium renale* or other diptheroid organisms; *Mycoplasma*-associated vulvovaginitis; and a condition of unknown aetiology. Other organisms associated with the disease that have been isolated from the lesions include *Streptococcus zooepidemicus* (Dunn 1996), *Histophilus ovis, Arcanobacterium (Trueperella) pyogenes, Mycoplasma fermentans* and *Mycoplasma bovigenitalium* (formerly *Mycoplasma* ovine/caprine serogroup 11) (Nicholas *et al.* 1998). The causative agent of ulcerative balanitis and vulvitis has not been conclusively identified and the aetiology of the disease has been ascribed to different infectious organisms by several researchers. A number of mollicutes, such as *M. bovigenitalium, Mycoplasma arginini, Mycoplasma mycoides* subsp*. mycoides* large colony variant (MmmLC), *M. mycoides* subsp. *capri (Mmc), Mycoplasma agalactiae, Mycoplasma capricolum, Acholeplasma laidlawii* and *Ureaplasma* species have been isolated from penile, preputial, vestibular vaginal and vulvar samples (Kidanemariam 2003).

Although it has been postulated that bacteria are the aetiological agents (Ball, Kennedy & Ellis 1991), some researchers regard caprine herpesvirus as the cause of the disease (Horner, Hunter & Day 1982), whilst according to others parapoxvirus (Linklater & Smith 1993) could possibly cause vulvovaginitis in sheep and goats, but their involvement in the pathogenesis of the disease needs to be established.

An MmmLC variant was isolated from several infected ewes and rams with vulvitis and balanitis in SA and inoculation of healthy animals with a field isolate reproduced the disease, which suggested that it may be the primary cause (Trichard *et al.* 1993). However, other organisms (*M. bovigenitalium, M. arginini, M. capricolum, A. laidlawii* and *Ureaplasma*) have been isolated in SA from sheep with the same clinical signs (Kidanemariam *et al.* 2005). MmmLC is a member of the *M. mycoides* cluster, a group of mycoplasmas that share serological, genomic and antigenic characteristics (Damassa, Wakenell & Brooks 1992). Although the MmmLC biotype is not associated with disease that is clinically and pathologically well defined, there are indications that this *Mycoplasma* could be involved in pathological conditions in small ruminants (Naglic *et al.* 2001). It has also been isolated from goats with polyarthritis, conjunctivitis, keratitis, pneumonia and cervical abscesses (Rosendal 1994).

Ulcerative vulvitis and balanitis in Dorper sheep started to receive serious scientific attention in SA during the last three decades. However, several *Mycoplasma* organisms recently isolated from cases of ulcerative balanitis in SA have been shown not to be MmmLC (Kimura 1980).

Diagnostic testing for members of the mycoides cluster proves difficult because of the similarities in clinical signs caused by each species, and the high degree of phenotypic and genetic similarity amongst them. In addition, intraspecies heterogeneity has been observed in MmmLC, Mmc and *Mycoplasma capricolum capricolum* (Mcc), whilst *M. mycoides* subsp*. mycoides* small colony (MmmSC) and *Mycoplasma capricolum capripneumonia* (Mccp) appear to be homogeneous. As a result of the diagnostic challenge, numerous polymerase chain reaction (PCR) assays have been developed based on various gene targets, such as CAP-21 (Bashiruddin, Taylor & Gould 1994), *M. mycoides* cluster (Rawadi, Lemercier & Roulland-Dussoix 1995), 16S rRNA (Bolske *et al.* 1996), lipoprotein gene (Monnerate *et al.* 1999) and insertion element (Van Kuppeveld *et al.* 1992). Real-time PCR (RT-PCR) assays have been developed that are highly sensitive and specific and provide accurate detection and differentiation of the members of the mycoides cluster (Fitzmaurice *et al.* 2008). Several of these assays require further analysis using restriction enzyme digestion or DNA sequencing and also post-PCR processing such as gel electrophoresis or southern blotting.

## Materials and methods

### Mycoplasma strains

Thirty-four *Mycoplasma* strains isolated from swabs and scrapings taken from the genital tract of Dorper sheep with clinical signs of ulcerative balanitis and vulvitis were included in this study. The samples were collected in 2003 from animals from 15 different farms covering five districts of the Northern Cape and Western Cape provinces of SA. Several additional strains that have been isolated in recent years from diagnostic samples submitted to the Faculty of Veterinary Science at the University of Pretoria were also included. The original samples, collected during 2003, were retrieved from storage and subjected to mycoplasmal isolation and purification procedures. Samples were originally catalogued and stored at −85 °C in Hayflick's medium in the bacteriology laboratory of the Department of Veterinary Tropical Diseases in the Faculty of Veterinary Science at the University of Pretoria, as previously described (Rosendal 1994; Ruhnke 1994).

A *Mycoplasma* reference strain (*M. mycoides* subsp. *mycoides* Y-goat 11706) and *Escherichia coli* strain S4 (09:K30) were obtained from the bacteriology laboratory of the Department of Veterinary Tropical Diseases. *Mycoplasma mycoides* subsp. *mycoides* Y-goat 11706 was used as a positive control and *E. coli* strain S4 (09:K30) and water were used as negative controls.

### Mycoplasma *growth conditions*

*Mycoplasma* strains were cultivated on Hayflick's agar medium (Ruhnke 1994) and then subcultured on corresponding broth medium. The presence of L-form bacteria was determined by inoculation of the broth cultures onto blood agar plates (Simecka *et al.* 1992). Bacterial colonies on blood agar plates were stained with Gram's stain and additional biochemical tests, such as growth on McConkey agar, catalase, oxidase, glucose fermentation and arginine utilisation, were performed on all of the samples using methods that have been described previously (Ernø & Stipkovits 1973).

### Nucleic acid-based analysis

Three millilitres of cultured broth were centrifuged at 6797 g for 10 minutes. The pellets obtained were subjected to two extraction methods, boiling and a kit method (Qiagen QiaAmp^®^ DNA mini kit, Whitehead Scientific, SA). The boiling method (Fan, Kleven & Jackwood 1995) entailed suspension of the pellet in 200 µL phosphate-buffered saline. This suspension was boiled at 96 ºC for 10 minutes, cooled on ice and centrifuged at 20 000 g for 2 minutes (Fan *et al.* 1995). The supernatant was collected and stored at −20 ºC until used in PCR assays.

The Qiagen, QiaAmp^®^ DNA mini kit was used according to the manufacturer's manual. Concentration determinations for both methods were done with a spectrophotometer (NanoDrop^®^ ND-1000, Thermo Fisher Scientific, Inqaba Biotechnical, Industries [Pty] Ltd, SA) and agarose gel electrophoresis (Rana, Gupta & Banga 1993). Extracted DNA was stored at −20 ºC until use.

PCR amplification was conducted using an upstream primer specific for the 16S rRNA (Rawadi *et al.* 1995) and a downstream primer specific for the genus *Mycoplasma* (Ruhnke 1994) (Table 1). An additional set of primers developed by Dr J. Picard (Department of Microbiology, James Cook University, Australia) and designated croc primers (Croc 1 and Croc 2) (Table 1), were also used. Both primer sets amplified a product of ~1078 base pairs (bp) in the 16S rRNA genome. The PCR was performed in a 25 µL reaction volume containing 12.5 µL Takara EX Taq^TM^ Premix (Takara Ex Taq^Tm^1.25 units/µL, dNTP mixture, 2× concentration each 0.4 Mm, EX Taq^TM^ buffer 2× including 4 mM Mg^2+^, Separations, SA); 0.5 µL of each oligonucleotide primer (Myco-forward and Myco-reverse; Croc 1 and Croc 2) (20 pM/µL) (Inqaba Biotechnical Industries [Pty] Ltd, Pretoria, SA) and 1 µL of the DNA (50 ng – 70 ng). The mixtures were subjected to 10 minutes of initial denaturation at 94 ºC, followed by 35 cycles of amplification involving denaturation at 94 ºC for 30 seconds, primer annealing at 59 ºC for 45 seconds, primer extension at 72 ºC for 45 seconds, and a final primer extension at 72 ºC for 7 minutes, using a DNA thermal cycler (Gene^Amp^ PCR system 9700, Applied Biosystems, SA).

**TABLE 1:** Primer sequences used for polymerase chain reaction and sequencing.

Number	Primer pairs	Oligonucleotide sequences (5′–3′)	Procedures performed	References
1	Myco-upstream (Croc1)	AGAGTTTGATCCTGGCTCAGGA	PCR sequencing	Robertson *et al.* (1993)
2	Myco-downstream	TGCACCATCTGTCACTCTGTTAACCC	PCR sequencing	Van Kupperveld *et al.* (1992)
3	FBAA5	GGAATATTGGACAATGGG	Sequencing	Weisburg *et al.* (1991)
4	RBAA5	GGAATATTGGACAATGGG	Sequencing	Weisburg *et al.* (1991)
5	FBAA6	GCGTGGGGAGCAAACAGG	Sequencing	Weisburg *et al.* (1991)
6	RBAA6	CCTGTTTGCTCCCCACGC	Sequencing	Weisburg *et al.* (1991)
7	FBAA7	ACGCGAAAAACCTTACC	Sequencing	Weisburg *et al.* (1991)
8	RBAA7	GGTAAGGTTTTTCGCGT	Sequencing	Weisburg *et al.* (1991)
9	FBAA8	GGAGGAAGGTGGGGA	Sequencing	Weisburg *et al.* (1991)
10	RBAA8	TCCCCACCTTCCTCC	Sequencing	Weisburg *et al.* (1991)
11	FBAA9	CGGTGAATACGTTCTCGGG	Sequencing	Weisburg *et al.* (1991)
12	RBAA9	CCCGAGAACGTATTCACCG	Sequencing	Weisburg *et al.* (1991)
13	pJET1.2/F	CGACTCACTATAGGGAGAGCGGC	Sequencing	pJET® 1.2 blunt cloning vector (Fermentas, Inqaba Biotechnology SA)
14	pJET1.2/R	AAGAACATCGATTTTCCATGGCAG	Sequencing	pJET® 1.2 blunt cloning vector (Fermentas, Inqaba Biotechnology SA)
15	Croc 2	GGTAGGGATACCTTGTTACGACT	PCR sequencing	Dr J. Picard, unpublished

Note: Please see the full reference list of the article, Kalshingi, H.A., Bosman, A-M., Gouws, J. & Van Vuuren, M., 2015, ‘Molecular characterisation of *Mycoplasma* species isolated from the genital tract of Dorper sheep in South Africa’, *Journal of the South African Veterinary Association* 86(1), Art. #1199, 11 pages. http://dx.doi.org/10.4102/jsava.v86i1.1199, for more information.

Amplified products were analysed together with a DNA ladder (O'Gene ruler^TM^, Fermentas Life Sciences, Inqaba Biotechnical, Industries [Pty] Ltd, Pretoria, SA) on a 1.5% agarose gel (Celtic Molecular Diagnostics, SA). Gels were stained with ethidium bromide, visualised and documented with Kodak electrophoresis documentation (EDAS, 290; Eastman Kodak Company, New York).

The QIAquick^®^ PCR Purification Kit protocol was obtained from Qiagen, Whitehead Scientific, SA and applied to purified PCR products before cloning them into pJET^®^ 1.2 cloning vector (Fermentas, Inqaba Biotechnical Industries [Pty] Ltd, Pretoria, SA), and the pGEM^®^T Easy vector system (Promega, Anatech, SA). The protocol of the pJET^®^ 1.2 system has been adapted to be the same as that for the pGEM^®^ T Easy vector system. Plasmid DNA was purified using the High Pure plasmid purification kit (Roche Diagnostics, SA) and subjected to sequencing and phylogenetic analysis (Inqaba Biotechnical Industries [Pty] Ltd, Pretoria, SA). Sequencing data were generated using primer pairs, as shown in Table 1, assembled and edited to a total length of 1078 bp using Gap 4 of the Staden package (Staden 1996). Sequencing data obtained were deposited in Gen Bank (http://www.ncbi.nih.gov).

Basic Local Alignment Search Tool (BLAST) searches of the sequences were conducted using the National Centre of Bioinformatics website to determine similarity between sequencing data obtained from local strains and those available in GenBank. Data were recorded as percentage similarity to related species.

Similarity matrices were constructed from six species, namely *Mycoplasma arginini, M. bovigenitalium, A. laidlawii, Mycoplasma* sp. ovine/caprine serogroup II*, Mycoplasma canadense* and MmmLC, using the double-parameter model (Kidanemariam *et al.* 2005) and the Jukes and Cantor correction model for multiple base changes (Jukes & Cantor 1969). Phylogenetic trees were constructed using neighbour joining (Saito & Nei 1987) and the maximum parsimony methods using the Mega 3.0 software package (Kumar, Tamura & Nei 2004). This was used in combination with the bootstrap method (Felsenstein 1985) (1000 replicates/tree for distance methods and 100 replicates tree for parsimony methods).

## Results

### Bacteriological analysis

All 34 isolates obtained following culture on Hayflick's agar yielded negative results with Gram's staining and were negative on the catalase and oxidase tests. Eighteen isolates (18/34) hydrolysed arginine and 14 (14/34) were glucose positive. Two (2/34) organisms (B1/01; B3/01) were not able to hydrolyse arginine or ferment glucose. All the isolates (*n* = 34) were tested for L-forms of bacteria by inoculating them onto blood agar. No bacterial growth was observed, and biochemistry results were concordant with the findings obtained with molecular methods.

### Nucleic acid analysis

**FIGURE 1:**
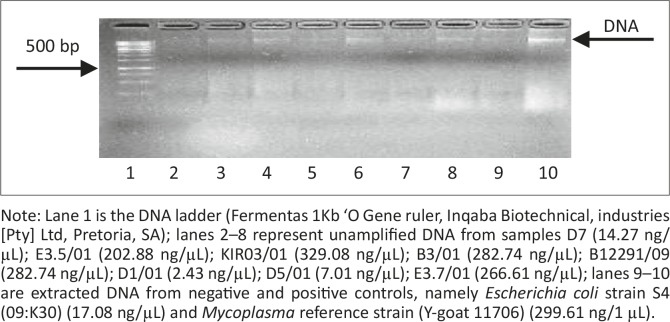
Electrophoretic analysis of unamplified DNA on a 1% agarose gel. Note: Lane 1 is the DNA ladder (Fermentas 1Kb ‘O Gene ruler, Inqaba Biotechnical, industries [Pty] Ltd, Pretoria, SA); lanes 2–8 represent unamplified DNA from samples D7 (14.27 ng/µL); E3.5/01 (202.88 ng/µL); KIR03/01 (329.08 ng/µL); B3/01 (282.74 ng/µL); B12291/09 (282.74 ng/µL); D1/01 (2.43 ng/µL); D5/01 (7.01 ng/µL); E3.7/01 (266.61 ng/µL); lanes 9–10 are extracted DNA from negative and positive controls, namely *Escherichia coli* strain S4 (09:K30) (17.08 ng/µL) and *Mycoplasma* reference strain (Y-goat 11706) (299.61 ng/1 µL).

DNA was successfully extracted from all samples (*n* = 34) and amplified using the procedures described in the ‘Materials and methods’ section. Extracted DNA was tested for purity and concentration prior to amplification by means of gel electrophoresis (Figure 1) and spectrophotometry. DNA concentrations ranged from 0.71 ng/µL to > 266 ng/µL and 50 ng – 70 ng of DNA was subsequently used in 25 µL of the PCR mixture.

**FIGURE 2:**
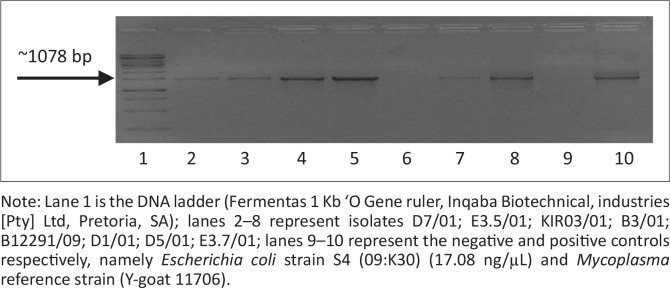
Polymerase chain reaction products generated with Croc primers on a 1% agarose gel. Note: Lane 1 is the DNA ladder (Fermentas 1 Kb ‘O Gene ruler, Inqaba Biotechnical, industries [Pty] Ltd, Pretoria, SA); lanes 2–8 represent isolates D7/01; E3.5/01; KIR03/01; B3/01; B12291/09; D1/01; D5/01; E3.7/01; lanes 9–10 represent the negative and positive controls respectively, namely *Escherichia coli* strain S4 (09:K30) (17.08 ng/µL) and *Mycoplasma* reference strain (Y-goat 11706).

**FIGURE 3:**
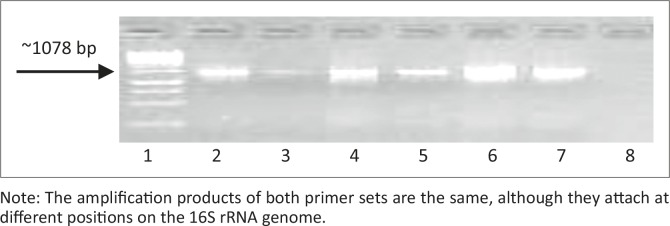
Illustrates the amplification product using Myco-upstream and Myco-downstream primers (Table 1). Note: The amplification products of both primer sets are the same, although they attach at different positions on the 16S rRNA genome.

The first amplification attempts using primer set Myco-upstream (Croc 1) and Myco-downstream (Table 1, Figure 2), resulted in low yields of amplification products. Primer set Myco-upstream Croc 1 and Croc 2 was applied (Figure 3) and resulted in better yields of amplification products. Products obtained using Croc 1 and Croc 2 were used in cloning and sequencing assays.

Initially DNA from all 34 isolates was extracted, amplified and directly sequenced. The primer set Croc 1 and Croc 2 was used in the sequencing reaction. Good sequence data could only be obtained from 22 isolates, and the decision was made to clone the PCR products for the remaining 12 isolates. The PCR products were cleaned and concentration determinations were done by means of gel electrophoresis (Figure 1) and spectrophotometry before cloning.

**FIGURE 4:**
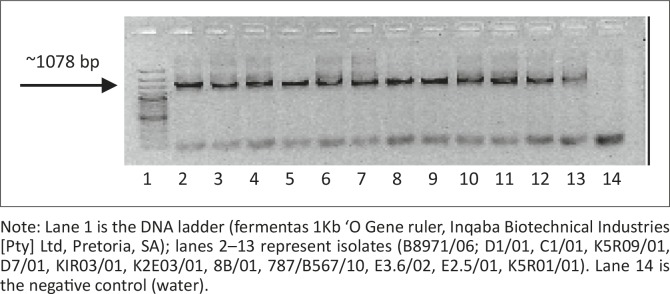
Polymerase chain reaction amplification products (4 reactions per isolate) on a 1% agarose gel for downstream applications. Note: Lane 1 is the DNA ladder (fermentas 1Kb ‘O Gene ruler, Inqaba Biotechnical Industries [Pty] Ltd, Pretoria, SA); lanes 2–13 represent isolates (B8971/06; D1/01, C1/01, K5R09/01, D7/01, KIR03/01, K2E03/01, 8B/01, 787/B567/10, E3.6/02, E2.5/01, K5R01/01). Lane 14 is the negative control (water).

**FIGURE 5:**
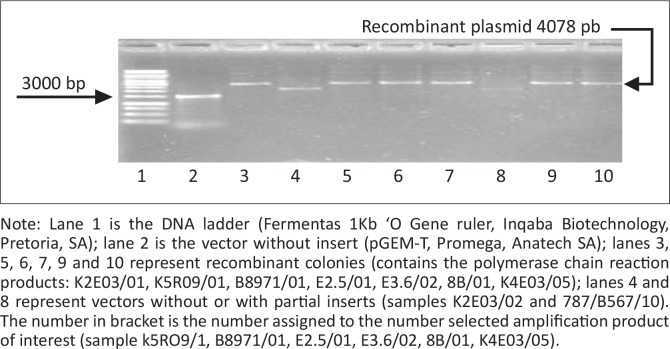
Purified plasmids. Note: Lane 1 is the DNA ladder (Fermentas 1Kb ‘O Gene ruler, Inqaba Biotechnology, Pretoria, SA); lane 2 is the vector without insert (pGEM-T, Promega, Anatech SA); lanes 3, 5, 6, 7, 9 and 10 represent recombinant colonies (contains the polymerase chain reaction products: K2E03/01, K5R09/01, B8971/01, E2.5/01, E3.6/02, 8B/01, K4E03/05); lanes 4 and 8 represent vectors without or with partial inserts (samples K2E03/02 and 787/B567/10). The number in bracket is the number assigned to the number selected amplification product of interest (sample k5RO9/1, B8971/01, E2.5/01, E3.6/02, 8B/01, K4E03/05).

A total of 120 plasmid colonies from 12 samples were screened for recombination by gel electrophoresis (Figure 4) and amplification using Croc 1 and Croc 2 primers (Figure 5). Only recombinant plasmids were further analysed. A total of 12 from 120 colonies were sequenced.

### Phylogenetic analysis

**FIGURE 6:**
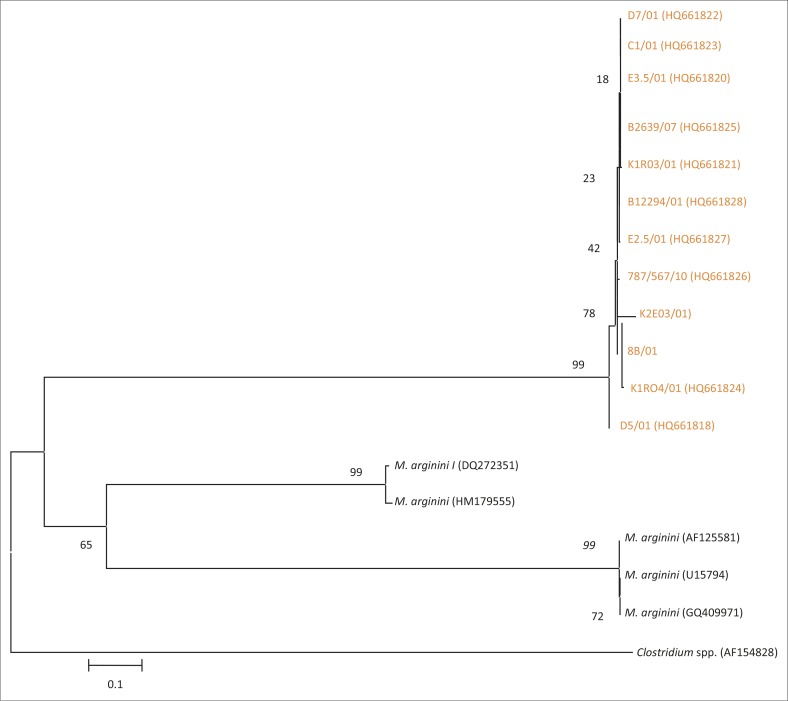
Results of the neighbour-joining analysis of the 16S rRNA gene showing the phylogenetic relationship of *Mycoplasma* field-isolate sequences with *Mycoplasma arginini* sequences collected from GenBank. *Clostridium* spp. (AF154828) was used as an out-group.

**FIGURE 7:**
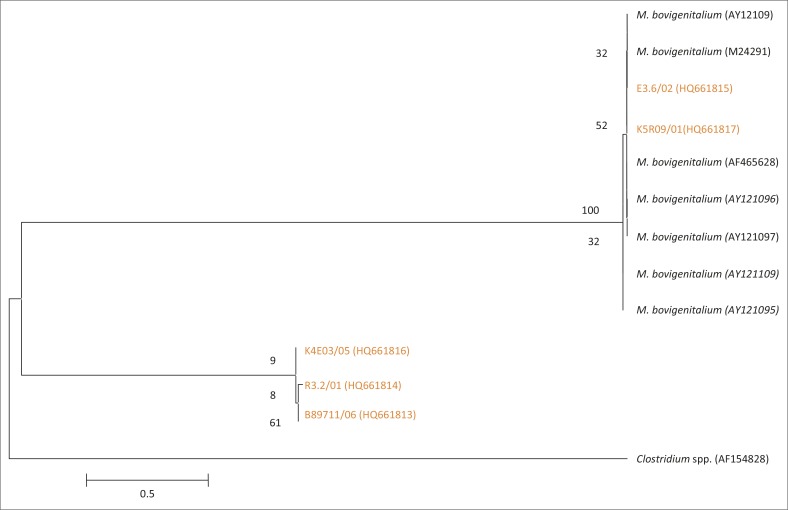
Phylogenetic tree based on the 16S rRNA gene sequences of five field isolates of *Mycoplasma bovigenitalium* from SA highlighted in orange and seven reference strains from Genbank. AF154828, a *Clostridium* spp., was used as an out-group.

**FIGURE 8:**
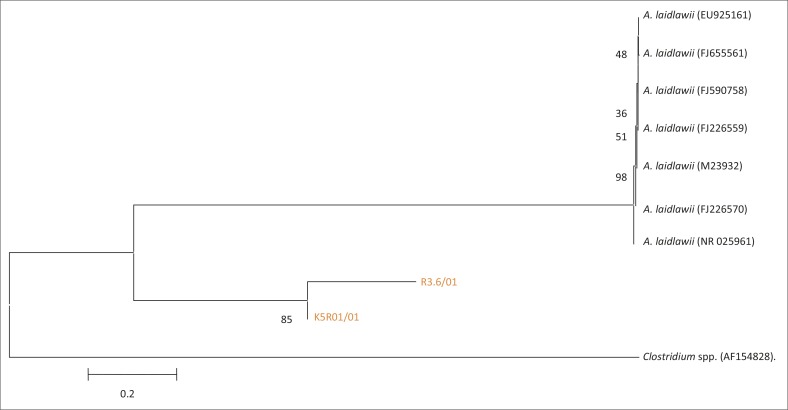
Phylogenetic tree based on the 16S rRNA gene sequences of two field isolates of *Acholeplasma laidlawii* from SA highlighted in orange, and seven reference strains from Genbank. AF154828, a *Clostridium* spp., was used as an out-group.

**FIGURE 9:**
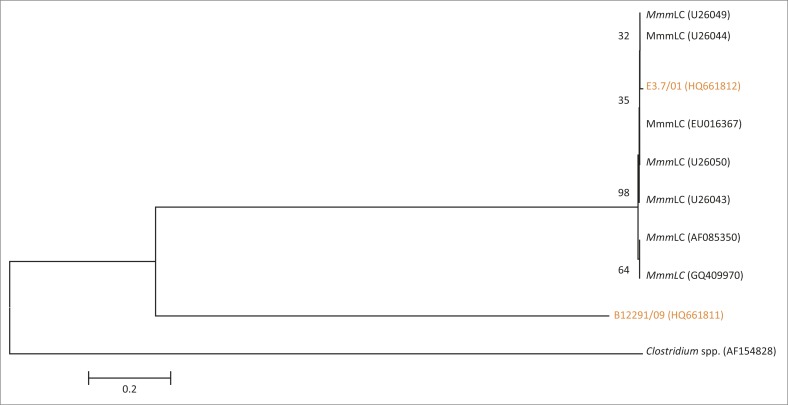
Phylogenetic tree based on the 16S rRNA gene sequences of two isolates of MmmLC from SA highlighted in orange and seven reference strains from Genbank and *Clostridium* spp. as out-group.

**FIGURE 10:**
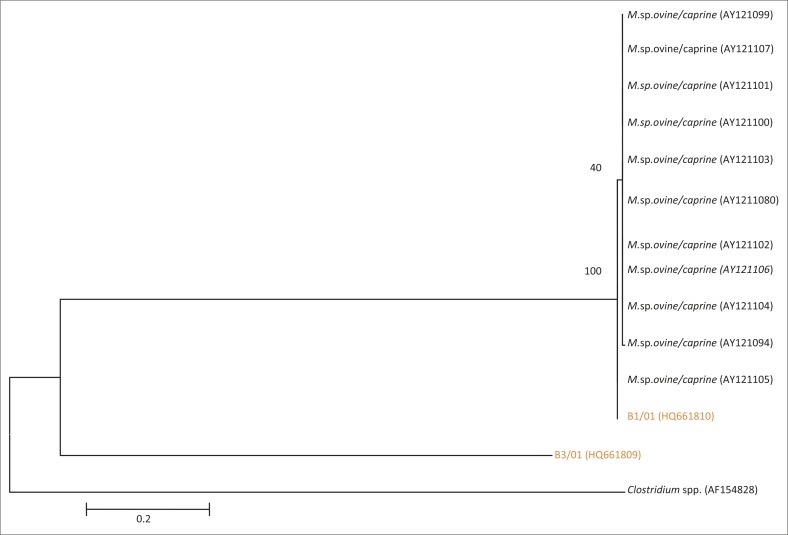
Phylogenetic tree based on partial 16S rRNA gene sequences of two field isolates of *Mycoplasma* sp. ovine/caprine serogroup II from SA highlighted in orange and 11 Genbank reference strains. AF154828, a *Clostridium* spp., was used as an out-group.

**FIGURE 11:**
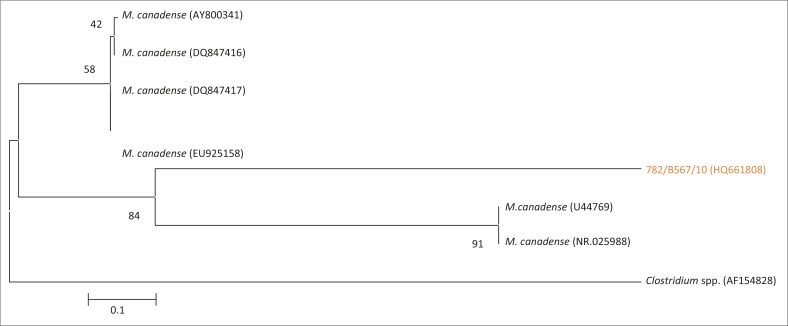
Phylogenetic tree based on the partial 16S rRNA gene sequence of one field isolate of *Mycoplasma canadense* from SA highlighted in orange, andsix *Mycoplasma canadense* reference strains from Genbank. AF154828, a *Clostridium* spp., was used as an out-group.

BLAST results revealed six distinct *Mycoplasma* species, namely *M. arginini, M. bovigenitalium, A. laidlawii*, MmmLC, *Mycoplasma* sp. ovine/caprine serogroup II and *M. canadense*. Sequence data representing each of the 34 isolates were further analysed using phylogenetic assays (Figures 7–11). BLAST results revealed that 18 of 34 isolates (50%) were *M. arginini* with 99% – 100% similarity to previously published *M. arginini* 16S rRNA gene sequences from England and Sweden (GQ409971 and AF125581). Phylogenetic analysis of the *M. arginini* group of sequences showed that all those obtained from isolates D7/01, C1/01, E3.5/01, B2639/07, K1R03/01, B12294/01, E2.5/01, 787/B567/10, K2E03/01, 8b/01, K1R04/01 and D5/01 were highly similar to each other but branched separately from sequences obtained from GenBank (Figure 6).

Six isolates (K5R09/01; R3.2/01; B8971/06; B8973/06; E3.6/02; K4E03/05) showed a high similarity to *M. bovigenitalium* (99% similarity to *M. bovigenitalium* from Turkey and Germany AF464628 and AY121098). Two isolates (B1/01, B3/01) were found to have a 99% identity to *Mycoplasma* sp. ovine/caprine sero group II sequences from the UK (AY121104). Phylogenetic analysis of these BLAST results (Figure 7) showed that two isolates (E3.6/02; K5R09/01) had a high similarity to GenBank sequences AY12109, M24291, AF465628, AY121096, AY121097, AY121109, and AY121095, whilst isolates K4EO3, R3.2 and B89711 branched separately.

BLAST results of two isolates (R3.6/01, K5R01/01) revealed a high similarity (99%) to *A. laidlawii* from India (FJ655561). Phylogenetic analysis showed that these two isolates grouped separately from GenBank sequences (Figure 8).

BLAST results revealed that isolates E3.7/01 and B12291/09 had a similarity of 99% to MmmLC (U26049) from Sweden. Isolates R3.4/02 and B12296/09 showed a similarity of 98% to *M.* sp. Usp 120 partial 16S rRNA gene sequences from Brazil (GU227399). BLAST searches for the two isolates K2E01 and B1857 showed similarity of 97% – 99% to Mmm Jcv1 (CP002027) partial 16S rRNA gene sequences from the United States of America (USA). Phylogenetic analysis (Figure 9) revealed that E.3.7/01 clustered together with GenBank sequences U26049, U26044, GQ409970 and AF085350 from Sweden, England, Mexico and France, whilst B12291/09 branched on its own.

Phylogenetic analysis showed B1/01 in monophyletic group with GenBank sequences AY121094 from UK of *Mycoplasma* sp. ovine caprine serogroup II, whilst B3/01 branched on its own (Figure 10).

BLAST results showed that isolate 782/B567/10 had a similarity of 99% to *M. canadense* partial 16S rRNA gene sequence data from Italy (NR025988). This isolate formed a monophyletic group with U44769 and NR025988 from the USA and Sweden in the phylogenetic analysis (Figure 11).

## Discussion

Infections with mycoplasmas are commonly associated with mucous and serosal membranes of the respiratory tract, urogenital tract, mammary gland, joints and eyes. Although mycoplasmas are largely host-specific, closely related animal species may share the same species. *Mycoplasma, Ureaplasma* and *Acholeplasma* spp. have been isolated from the urogenital tract and organs of healthy sheep and from sheep with clinical signs of balanoposthitis and vulvovaginitis. Although the aetiology of the latter still remains unresolved, it is considered to be multifactorial.

Evidence suggests that *Mycoplasma, Ureaplasma* or *Acholeplasma* infections are the primary causal factors in ulcerative balanitis and vulvitis, whilst end-stage bacterial pathogens such as *Trueperella pyogenes* are responsible for most of the lesions observed. Some authors have also incriminated viruses, but little support for their role in the pathogenesis of ulcerative balanitis and ulcerative vulvitis has been published (Kidanemariam *et al.* 2005).

In this study a total of 34 isolates from clinical cases of ulcerative vulvitis and balanitis were successfully cultivated on Hayflick's medium and identified and characterised. Bacteriological analysis of all the isolates was done by assessment of growth on Hayflick's agar, morphological appearances and biochemical tests. PCR and sequencing of the partial 16S rRNA gene was used for the first time during this project to characterise *Mycoplasma* spp. involved in ulcerative balanitis and vulvitis in sheep in SA. The PCR products that were sequenced did not yield good results for eight isolates with the identity numbers K2EO3/01, B8971/06, E3.6/01, K5R09/01, 8B/01, E2.5/01, 787/B567/01 and K4E03/05. It was therefore decided to clone them and sequence the recombinant plasmids with the inserts. Results obtained from the conventional methods of detection and characterisation were in agreement with the molecular methods of identification used.

The results showed that 18 isolates (51%) were strains of *M. arginini*. The isolation and identification of *M. arginini* from cases of ulcerative vulvovaginitis and balanoposthitis in goats and sheep in SA and Nigeria have been reported (Chima & Ojo 1986; Greig 2007; Kidanemariam 2003). It was also previously isolated from cases of ovine kerato conjunctivitis and mastitis.

Molecular analysis through BLAST searching of the 18 isolates showed 99% – 100% similarity with previously published sequences of *M. arginini* 16S rRNA genes submitted to GenBank that were isolated in England and Sweden (Weisburg 1991).

Kidanemariam (2003) isolated four *M. arginini* strains from 104 sheep affected with ulcerative balanitis and ulcerative vulvitis in SA and 116 unaffected sheep. In this study 18 *M. arginini* strains from 34 isolates from cases of ulcerative balanitis and ulcerative vulvitis were identified by both conventional and molecular methods. A high number of isolates of this organism does not confirm it to be the causative agent for the disease, since the organism is known to have low pathogenicity in animals even though it has been isolated from cases of mastitis, pneumonia, arthritis and reproductive disease. Experimental animal studies done with this organism to prove it played a role in causing pneumonia have been published (Jones *et al.* 1983). The conclusion was that it was not capable of predisposing the lung to secondary invasion by *Mannheimia haemolytica*, or of exacerbating the pneumonia. The current findings highlight the need to re-examine the possible role that strains of *M. arginini* can play in diseases of ruminants.

Of the 34 isolates characterised, two strains (B12291/09 and E.3.7/01) previously isolated from cases of ulcerative balanitis and vulvitis in Dorper sheep tested negative for arginine and were negative on other biochemical tests but positive for glucose. They also showed sequence similarity of 99% with an MmmLC strain that was isolated from a domestic goat (*Capra hircus*) in Sweden (strain UM32847). Trichard *et al.* (1993) inoculated sheep intravaginally using an MmmLC isolate to try and establish the aetiology of ulcerative balanitis and vulvitis in Dorper sheep in SA. The authors re-isolated the same organism from the diseased animals, and concluded that MmmLC is the causative agent of ulcerative balanitis and vulvitis. This finding was supported by Kidanemariam (2003), who obtained 104 strains of MmmLC from 220 samples collected from Dorper sheep on different farms in SA. However, both studies only made use of conventional identification methods.

In view of the results of previous South African studies, it was surprising that so few strains of MmmLC were detected, even though the numbers of isolates were relatively small. This may suggest that it is not the primary cause of ulcerative balanitis and ulcerative vulvitis. The high number of strains reported in the study by Kidanemariam (2003) may point to an over-estimation of the possible role of MmmLC as a result of serological cross-reactions in the fluorescent antibody test, which have been documented for several serological assays.

Five isolates gave positive reactions for glucose but were negative for all other biochemical tests. These were identified as *M. bovigenitalium* and revealed a sequence similarity of 99% with *M. bovigenitalium* isolated from cattle in Germany. The isolation of *M. bovigenitalium* is in accordance with earlier isolations from goats and sheep that suffered from a similar disease (Chima & Ojo 1986; Kidanemariam 2003). *Mycoplasma bovigenitalium* is also known to be the cause of a similar problem in cattle. Saed and Al-Aubaid (1983) confirmed the pathogenicity of *M. bovigenitalium* by experimentally inseminating 12 heifers with it; the entire group developed granular vulvovaginitis. Similarly, Broughton, Hopper and Gayford (1983) reported isolation of *M. bovigenitalium* and *M. canadense* from an outbreak of granular vulvovaginitis in Israeli dairy herds (Broughton *et al.* 1983). Afshar and Fabricant (1971) confirmed the pathogenicity of this organism in granular vulvovaginitis in cattle. These findings have further confirmed that *M. bovigenitalium* was a cause of genital mycoplasmosis in cattle.

Further characterisation identified two isolates that were unable to ferment glucose or to hydrolyse arginine; these were confirmed by sequencing to be 99% similar to *Mycoplasma* sp. ovine/caprine serogroup II. The first isolations of *Mycoplasma* sp. ovine/caprine serogroup II from cases of ulcerative balanitis and vulvovaginitis were reported from sheep in Australia (Ruhnke 1994) and experimental infections in goats (Rana *et al.* 1993). Davidson and Stuart (1960) also reported the isolation of *M. bovigenitalium* that was very similar to *Mycoplasma* sp. ovine/caprine serogroup II, as neither fermented glucose nor hydrolysed arginine nor possessed phosphatase activity. According to Al-Aubaid *et al.* (1972) this isolate is biochemically similar to *M. bovigenitalium;* they share close genetic and phenotypic characteristics, with similar clinical manifestations observed in reproductive disorders in both cattle and small stock (Rosendal 1994; Saito & Nei 1987). These isolates shared serological relationships with the *mycoides* cluster. Appeals to classify these two organisms into a single species appeared in 2002, and were officially requested in a proposal published by Nicholas *et al.* (2008).

During this study two isolates that were previously isolated from cases of ulcerative balanitis and vulvovaginitis in sheep in SA were found to be *A. laidlawii*. This has been reported in sheep flocks in Australia and in free-living European bison (*Bison bonosus*) with balanophosthitis.

An interesting finding was two strains identified as *Mycoplasma* sp USP 120, which is the first isolation and characterisation of this organism from cases of ulcerative balanitis and vulvitis of Dorper sheep. These strains were found to be 99% similar to an organism (GU227399) from Brazil that was isolated from the urogenital tract of sheep (unpublished results, GenBank).

Of the 34 isolates, two were found to be similar to the synthetic *M. mycoides* JCVI-syn1.0 clone described by Gibson *et al.* (2010). However, it must be borne in mind that it was demonstrated that the flanking regions of the 5’ V3 region are highly conserved amongst prokaryotes, whilst the 3’ V3 are conserved amongst mycoplasmas and ureaplasmas (Yoshid *et al.* 2002). This can explain why the plasmid sequences R3.4/02 and B12296 showed a similarity to *M. mycoides* JCVI-syn1.0. Gibson *et al.* (2010) created new *M. mycoides* cells that are controlled by a synthetic chromosome. The only DNA in the cells is the designed synthetic DNA sequence, including ‘watermark’ sequences and other designed gene deletion and polymorphism and mutation acquired during the building process. The new cells have expected phenotypic properties and are capable of continuing self-replication. Although these authors used genetic material from *M. capricolum* and *M. mycoides,* no conclusions can be drawn in terms of the two South African isolates that were identified as *M. mycoides* JCV1-syn1.0. To determine the true identity, additional clones of those two specific isolates will have to be sequenced. Therefore the potential of these organisms to cause lesions in the genital tract of sheep is not known.

One field isolate was identified as *M. canadense*, which has not previously been documented in sheep in SA. Similarly, there was a report of isolation of *M. canadense* and *M. bovigenitalium* as the cause of outbreaks of ulcerative posthitis in cattle in Israel (Nicholas *et al.* 2008; Petit *et al.* 2008). *Mycoplasma canadense* was also isolated from an unusual form of vulvitis in an outbreak affecting several heifers soon after introduction to a feedlot in SA (Gibson *et al.* 2010).

### Practical implications

Implications of the findings of this study are that development of an antimycoplasmal vaccine for protection against ulcerative balanitis/ulcerative vulvitis will be a complicated task, as several species have been implicated in the pathogenesis of the disease. A further implication is that control of the disease will for the foreseeable future be dependent on treatment with antimicrobial drugs, which will necessitate regular bacterial isolation and testing for resistance to the drugs.

## Conclusion

In conclusion, PCR amplification of the 16S rRNA gene and cloning and sequencing applied during this study identified all 34 Mycoplasma species isolated from clinical cases of ulcerative balanitis and vulvitis in Dorper sheep in SA. The techniques also enabled the identification of new species or strains of *Mycoplasma* not previously been described in the region. Species of *Mycoplasma* not previously described in SA included strains closely related to synthetic *M. mycoides* JCVI-syn1.0 clone and *Mycoplasma* sp. USP 120.

The results from this study support the findings of other researchers that ulcerative balanitis and vulvitus of sheep is a multifactorial disease that may involve different species of mycoplasmas. However, it does not provide strong support for the findings of Trichard *et al.* (1993) and Kidanemariam (2003) that MmmLC is the primary cause of ulcerative balanitis and vulvitis of Dorper sheep in SA. Rather, it would seem that several *Mycoplasma* species can cause primary insults to the reproductive tract of small stock, and that the end-stage pathogens that are responsible for visible lesions are pathogenic bacteria, most notably *T. pyogenes* (Thiede *et al.* 2002).
